# Emerging applications of single-cell profiling in precision medicine of atherosclerosis

**DOI:** 10.1186/s12967-023-04629-y

**Published:** 2024-01-23

**Authors:** Huiling Lin, Ming Zhang, Mi Hu, Yangkai Zhang, WeiWei Jiang, Wanying Tang, Yuxin Ouyang, Liping Jiang, Yali Mi, Zhi Chen, Pingping He, Guojun Zhao, Xinping Ouyang

**Affiliations:** 1 Department of Physiology, Medical College, Institute of Neuroscience Research, Hengyang Key Laboratory of Neurodegeneration and Cognitive Impairment, University of South China, Hengyang, 421001 Hunan China; 2 Department of Physiology, School of Medicine, Hunan Normal University, Changsha, 410081 Hunan China; 3 Affiliated Qingyuan Hospital, Guangzhou Medical University (Qingyuan People’s Hospital), Qingyuan, 511518 Guangdong China; 4 Department of Organ Transplantation, Zhujiang Hospital, Southern Medical University, Guangzhou, Guangdong China; 5 Department of Clinical Pharmacology, Xiangya Hospital, Central South University, Changsha, Hunan China; 6 College of Physics and Optoelectronic Engineering, Shenzhen University, Shenzhen, China; 7 Department of Nursing, School of Medicine, Hunan Normal University, Changsha, 410081 Hunan China; 8 The Key Laboratory of Model Animals and Stem Cell Biology in Hunan Province, School of Medicine, Hunan Normal University, 410081, Hunan Changsha, China; 9 The Engineering Research Center of Reproduction and Translational Medicine of Hunan Province, School of Medicine, Hunan Normal University, 410081, Hunan Changsha, China

**Keywords:** Single-cell sequencing, Precision medicine, Atherosclerosis, Cellular heterogeneity

## Abstract

Atherosclerosis is a chronic, progressive, inflammatory disease that occurs in the arterial wall. Despite recent advancements in treatment aimed at improving efficacy and prolonging survival, atherosclerosis remains largely incurable. In this review, we discuss emerging single-cell sequencing techniques and their novel insights into atherosclerosis. We provide examples of single-cell profiling studies that reveal phenotypic characteristics of atherosclerosis plaques, blood, liver, and the intestinal tract. Additionally, we highlight the potential clinical applications of single-cell analysis and propose that combining this approach with other techniques can facilitate early diagnosis and treatment, leading to more accurate medical interventions.

Atherosclerosis is a chronic, progressive inflammatory disease that occurs in the arterial wall [1–3]. It is characterized by lipid accumulation, inflammation of the large and medium-sized artery walls, and endothelial dysfunction [4, 5]. The mechanisms underlying the occurrence, development, regression, and plaque rupture of atherosclerosis are associated with cellular heterogeneity and plasticity [6–10]. This situation emphasizes the importance of gaining a deeper understanding of the underlying biology of this disease, particularly in terms of the complexity of defining the molecular events and heredity of atherosclerosis.

The goal of precision medicine is to utilize big data, including clinical, lifestyle, genetic, and biomarker information, to tailor individualized medical care based on the specific circumstances of each individual [11]. Since the initial publication of the next-generation sequencing (NGS) study in atherosclerosis, the genetic landscape of atherosclerosis has been extensively characterized [12]. By detecting the DNA or RNA information of individual cells in atherosclerosis, NGS reveals cellular heterogeneity in tissues and provides more detailed and accurate genetic information, offering significant prospects for clinical diagnosis and precise treatment [13–17]. In this review, we describe the emerging single-cell sequencing technique and its application in atherosclerosis. These technologies have the potential to facilitate a deeper analysis of atherosclerosis, translate the data into clinical applications, and provide improved precision medical services for individuals with atherosclerosis.

## Analysis of genetic heterogeneity in atherosclerosis

**Fig. 1:**
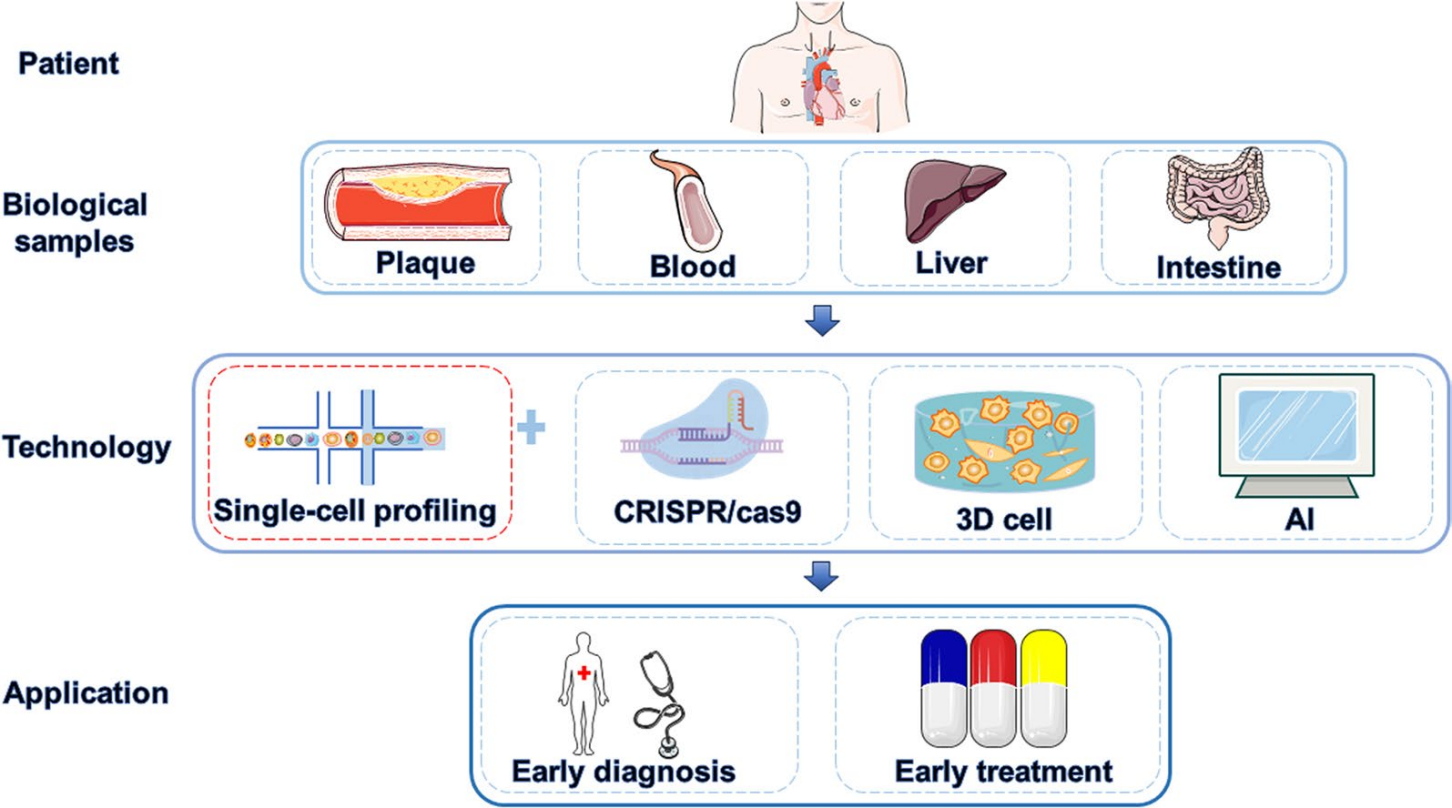
Scheme of Single-Cell Analysis in Precision Medicine for Atherosclerosis. The combination of single-cell analysis, CRISPR/Cas9 gene editing, three-dimensional cell (3D cell) analysis and artificial intelligence (AI) enables in-depth exploration of tissue heterogeneity in atherosclerosis plaques, blood, liver, and the intestinal tract. This approach can guide early diagnosis and treatment of atherosclerosis patients and achieve the goal of accurate medical treatment. By figdraw

**Fig. 2:**
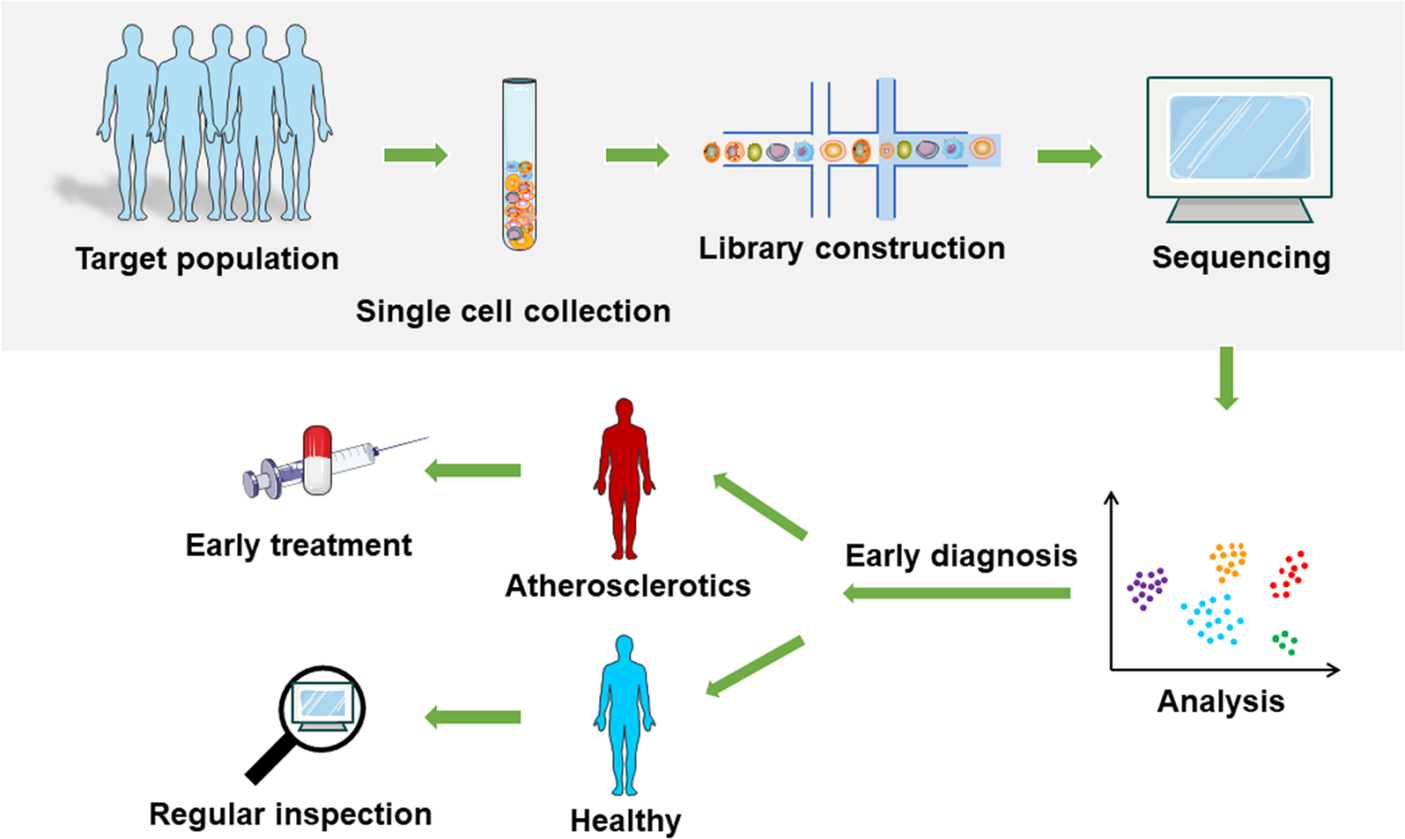
Graphics illustrate decision making procedure of atherosclerosis detection in the near future. Individual cells will be collected, followed by libraries constructing, sequencing and data analyzing. On the one hand, doctors can make early diagnosis of atherosclerotics and achieve the purpose of early treatment. On the other hand, healthy people can use single-cell sequencing to check regularly to achieve the purpose of early diagnosis

Atherosclerosis is a complex disease that has been characterized at the molecular level. Understanding the molecular identity of conserved gene expression in vascular components has provided novel insights into the management of atherosclerosis, enabling the identification of biomarkers for accurate diagnosis and genetic targets for personalized treatment. Over the past decades, the application of NGS has advanced our knowledge of the genetic landscape, revealing changes in genetic heterogeneity during the course of atherosclerosis. Genome-wide association studies (GWAS) combined with potentially implicated genes have served as unbiased tools for screening risk factors. To date, more than 200 genetic loci associated with the progression of chronic coronary artery disease [18–20] have been identified, including Recombinant DNA Methyltransferase 3A (DNMT3A), tet oncogene family member 2 (TET2), Additional sex comb-like 1 (ASXL1), Janus kinase 2 (JAK2), and others. Moreover, numerous clinical trials have demonstrated the value of investigating genetic heterogeneity. For instance, carriers of the GUCY1A1 gene may derive greater benefits from aspirin treatment, while interleukin expression has been shown to be a useful target in patients receiving canakinumab in the CANTOS trial. Therefore, translating genomic findings into specific solutions has the potential to optimize the diagnosis and treatment of atherosclerosis (Figs. 1 and 2).

Nevertheless, bulk RNA sequencing, which is the main technology of NGS, can only measure average gene expression, encompassing all individual cells and failing to discern heterogeneity between cells. However, atherosclerosis involves multiple cellular populations from blood and tissues, each playing a distinct role with different phenotypes and fates. For instance, clusters of macrophages can adopt either plaque-destabilizing or plaque-stabilizing phenotypes. Therefore, capturing the genetic heterogeneity within different cellular clusters is a critical aspect that has eluded researchers. Importantly, single-cell analysis overcomes these limitations of traditional sequencing methods. By assessing RNA expression at a single-cell resolution, transcriptome levels can be distinguished among cellular clusters, enhancing the sensitivity and accuracy of detection. Single-cell sequencing at different time points reveals the dynamic changes in genetic heterogeneity over time and enables the identification of cellular phenotypes. Given its recent remarkable advancements, single-cell sequencing has become an indispensable tool in atherosclerosis research.

## Single-cell sequencing in atherosclerosis

### Research progress of single cell sequencing

The rapid development of single-cell sequencing provides a new perspective for the study of single-cell characteristics of atherosclerosis. Bulk sequencing is a high-throughput sequencing technology suitable for large-scale genome research. It not only has less sample limitation [21], but also can quickly and efficiently detect biological information such as genome variation, gene expression and DNA methylation [22]. However, the data obtained by bulk sequencing is only the average or representative data of the gene expression of the cell population [23]. They cannot accurately reflect the specific situation of each subpopulation of cell due to the high heterogeneity between cells or the microenvironment around them [24]. Thus, this hinders a full understanding of risk stratification in disease and precise application of drug therapies [16, 17, 25, 26]. In contrast, single-cell sequencing enables high-resolution detection of individual cells, revealing cell heterogeneity, cell type composition, gene expression profiles, and more [24, 27, 28]. This approach provides a more detailed and direct assessment of unbiased samples for atherosclerosis bioinformatics studies.

So far, the development of single-cell sequencing has followed a common workflow, including sample preparation, single-cell capture, reverse transcription and amplification, library preparation, sequencing, and analysis [24]. Successful single-cell transcription data generation relies on effective single-cell sorting. In the context of atherosclerosis, solid tissue is digested and dissociated into single-cell suspensions, while peripheral blood samples are processed using enzymatic methods [29]. In the laboratory, techniques such as magnetically activated cell sorting (MACS), fluorescence-activated cell sorting (FACS) [28], or micro-droplet reaction technology can be employed to efficiently separate and retrieve cells before single-cell retrieval and library preparation for sequencing [30].

Currently, many new single-cell sequencing technologies are characterized by high-throughput capabilities and cost-effectiveness. These include the Fluidigm C1 and BD Rhapsody systems based on micropore technology, the WaferGen ICELL8 single-cell system utilizing microfluidic chip technology, the Single Cell 3 Solutions by 10X Genomics, and the DNBeLab C4 platform developed by Huada Intelligence. These advancements have increased processing capacity and reduced costs for high-throughput single-cell sequencing, thereby fostering the growth of a high-throughput, multi-cell, user-friendly single-cell sequencing industry in atherosclerosis [31].

### Insights from atherosclerosis plaque samples

Single-cell sequencing studies have demonstrated the extensive heterogeneity of atherosclerosis, revealing the transcriptome-based cellular landscape of human atherosclerotic plaques. These studies shed light on cellular plasticity and intercellular communication within disease sites[26], enhancing our understanding of the diverse cellular heterogeneity within the plaque environment. Single-cell sequencing allows a shift from a macroscopic perspective of atherosclerosis plaques to a focused cellular view, facilitating the discovery of novel therapeutic targets and advancing precision medicine for atherosclerosis.

Single-cell RNA and single-cell ATAC sequencing analyses of human carotid atherosclerotic plaques have identified 14 distinct cell groups, including endothelial cells, smooth muscle cells, mast cells, B cells, bone marrow cells, and T cells [26]. These cells can be categorized into immune cells and non-immune cells. Cochain et al. [16] conducted an analysis of aortic single-cell RNA sequencing data from atherosclerosis patients, identifying immune cell profiles consisting of three macrophage subsets and monocyte-derived dendritic cells. They also observed the expression of some uncharacterized trigger receptors, such as bone marrow cell 2 (TREM2), in macrophages. TREM2^+^ macrophages were predominantly observed in the cells of diseased aortas, providing new evidence for exploring this unique subset of macrophages and their function in atherosclerosis. Besides, Wang et al. [32] observed that the complex pattern of deterioration of peripheral T cell tolerance was most pronounced in plaques, and that human femoral plaques showed a significant decrease in macrophage spectrum and CD8^+^ T cell population compared with carotid plaques [33]. Vascular smooth muscle cells (VSMCs) are non-immune cells that participate in all stages of atherosclerotic plaque development. Pan et al. [17] integrated single-cell genomics with a VSMC taxonomic map and discovered that, in both mouse and human atherosclerotic plaques, VSMCs undergo a phenotypic transition into intermediate cell states called "SEM" cells. Their findings demonstrated that "SEM" cells were enriched with known differentiation markers of stem cells, endothelial cells, and monocytes/macrophages, suggesting a regulatory role in the phenotypic transformation of smooth muscle cells. This discovery presents a potential therapeutic target for the treatment of atherosclerosis (Table 1).

**Table 1:** Single-cell studies of different atherosclerosis samples in human and mice

Sample type	Species	Source	Methods	Major findings	References
Plaque	Human	Immune cells VSMCs Endothelial cells	scRNA-seq, scATAC-seq	The cellular landscape of human atherosclerotic plaques; cellular plasticity and intercellular communication at the site of disease	[26]
	Human	Macrophages CD4^+^ T cell CD8^+^ T cell	CyTOF CITE-seq scRNA-seq	Novel immune dysregulation in plaques associated with the clinically symptomatic disease T cell subsets present markers of T cell exhaustion macrophages contain alternatively activated phenotypes	[43]
	Human/Mice	Immune cells, VSMCs	CITE-seq scRNA-seq	The transcriptome phenotype of SMCs in mouse and human atherosclerosis lesions is comprehensively characterized TCF21 expression is closely associated with the regulation of SMC phenotype in diseased human coronary arteries	[89]
	Mice	Macrophages	scRNA-seq	Prophagocyte single-walled carbon nanotubes reduce inflammatory gene expression in diseased macrophages	[117]
	Mice	CD4^+^ T cells	scRNA-seq	Identification of CD4^+^ T cells that recognize apolipoprotein (apo) B in mice	[118]
	Mice	Immune cells	CyTOF scRNA-seq	The white blood cells from the aorta of healthy and atherosclerotic mice were deeply characterized; to determine the map of the immune cell landscape in atherosclerosis	[35]
	Mice	Macrophages	scRNA-seq	Heterogeneity of macrophages during the progression and regression of atherosclerosis; stem cell-like characteristics of monocytes	[119]

Blood	Mice	VSMCs	scRNA-seq	Heterogeneity of VSMCs in blood vessels of healthy mice; characteristics of disease-related transcription in VSMCs lineage cells	[120]
	Human	Monocyte	scRNA-seq	Humans have significant monocyte heterogeneity	[42]
	Human	Red blood cells	scRNA-seq	RBC RNA-Seq capture; transcriptional heterogeneity of red blood cells	[41]
	Human	Exosome Macrophage	scRNA-seq	Revealed the unique relationship between the transcription characteristics of macrophages and phenotypic heterogeneity in the microenvironment of atherosclerosis	[44]
	Human	PBMCs	CITE-seq	The practicality of scRNA seq in evaluating cell surface phenotype in the same cell	[36]
	Human	CD4^+^ T cells	scRNA-Seq, CITE-Seq	Gender differences in gene expression of CD4^+^ cells	[46]
	Mice	Adipocytes	scRNA-seq	Changes in adipocyte function are the basis for observed changes in plasma lipids	[45]
Liver	Human	Liver cells	scRNA-seq	Map of the human liver immune microenvironment	[62, 63]
	Mice	Adipocytes	scRNA-seq	Changes in adipocyte function are the basis for observed changes in plasma lipids	[45]
	Mice	Myeloid cells	scRNA-seq	Heterogeneity of bone marrow cells in the liver and bone marrow during NAFLD; NAFLD has driven in vitro macrophage functional adaptation and its functional correlation during in vivo steatohepatitis	[64]

Intestine	Mice	Intestinal flora	16S ribosomal RNA gene sequencing	Bile acid has been identified as an important metabolic factor related to the gut microbiota	[68]
	Mice	Fecal microorganisms	16S ribosomal RNA gene sequencing	PSRC1 participates in regulating the gut microbiome	[69]

*scRNA-seq* single-cell RNA sequencing, *scATAC-seq* single-cell Assay for Transposase-Accessible Chromatin using sequencing, *CyTOF* Cytometry by Time-of-Flight; CITE-seq, Cellular indexing of transcriptomes and epitopes by sequencing, *TCF21* transcription factor 21, *CX3CR1* fractalkine receptor, *VSMCs* Vascular smooth muscle cells, *PBMCs* Peripheral Blood Mononuclear Cells, *CAD* Coronary Artery Disease, *CVAV* Cardiovascular Assessment Virginia, *Trib1* tribbles homolog 1, *NAFLD* Non-alcoholic fatty liver disease, *PSRC1* Proline/serine-rich coiled-coil protein

In addition, the identification of stable and unstable plaques in atherosclerosis plays a crucial role in identifying patients at risk. Furthermore, understanding the cellular communication within plaques is vital for the maintenance of atherosclerotic plaques. Bao et al. [34] conducted next-generation RNA sequencing to analyze the transcriptomic profile of stable and unstable atherosclerotic plaques in human carotid arteries. They identified 202 mRNAs, 488 long noncoding RNAs, and 91 circular RNAs. On one hand, genes associated with plaque stability were found to be involved in functions related to immune response, neurological function, hematological activity, and endocrine system synthesis and secretion. On the other hand, unstable plaques exhibited the upregulation of five key genes: CD5L, S100A12, CKB, CEIP, and SH3GLB1. These genes were associated with M2 polarization, inflammation promotion, epithelial-mesenchymal transition, apoptosis, and autophagy, respectively. These findings highlight the importance of cell communication in maintaining atherosclerotic plaques [34].

Of note, there are limitations in the current detection methods for clinical coronary plaque samples. On one hand, laser capture microdissection is the only technique available to isolate specific regions of the plaque[35], but it does not yield a sufficient number of cells for single-cell sequencing. On the other hand, the prolonged digestion and mechanical disruption of frequently calcified human plaques may result in the selective loss of certain more vulnerable cell subpopulations, leading to incomplete data. However, there are approaches that can be employed to address these challenges. Unbiased amplification of DNA can be utilized to increase the genetic material content of a single cell, allowing for further sequencing. Moreover, integrating labeled gene transcripts (mRNA), labeled expression (proteins), and pathways (concentration of differentially expressed genes) can enhance the sensitivity of cell population detection, effectively circumventing these problems.

### Insights from blood samples

Non-invasive assessment of peripheral blood biomarkers for atherosclerosis holds great promise as a research direction [36]. Peripheral blood consists primarily of three types of blood cells, including complete white blood cells that can be captured for multi-omics analysis of DNA, RNA, and protein content. Moreover, it has been demonstrated that human red blood cells contain abundant and diverse microRNA and mRNA transcripts, making them suitable for single-cell sequencing [37–41]. A recent study utilizing single-cell sequencing of peripheral blood samples identified three subsets of CD16 nonclassical monocytes and four subsets of CD14 classical monocytes within peripheral blood mononuclear cells (PBMCs) from 18 atherosclerosis patients [42]. Additionally, seven distinct clusters of erythrocytes were identified at the single-cell level, successfully capturing the heterogeneity of atherosclerosis in liquid biopsy samples [41]. Interestingly, CD14 monocytes (MC13), natural killer (NK) cells (MC6), plasmacytoid dendritic cells (PDC) (MC16), and B cells (MC3) were found to be more abundant in blood compared to plaques [43]. These findings suggest that blood may serve as an alternative to plaque samples for cellular analysis of atherosclerosis in patients, enabling risk stratification and precision medicine based on simple blood draws.

Single-cell sequencing of peripheral blood samples from individuals with atherosclerosis not only enables the identification of specific cell subsets but also provides new insights into the pathogenesis of the disease, allowing for a comprehensive understanding of its etiology and risk factors. For instance, Li and colleagues conducted RNA sequencing analysis of exosomes extracted from the plasma of patients with coronary artery disease (CAD) and discovered that miR-4498 from plasma exosomes can inhibit the expression of inflammatory cytokines such as Compatible Time-Sharing System (Ctss) and triggering receptor expressed on myeloid cells 2 (Trem2) [44]. In another study, Elizabeth et al. [45] performed RNA-seq of adipocytes from Trib1_ASKO mice and revealed that Tribbles homolog 1 (Trib1) is a crucial regulator of adipocyte function. These findings significantly enhance our understanding of atherosclerosis.

Indeed, the application of single-cell sequencing analysis to peripheral blood samples from individuals with atherosclerosis demonstrates its potential for clinical use. Ryosuke and colleagues conducted a comprehensive analysis of CD4^+^ cells from 61 human peripheral blood samples. The findings revealed significant gender differences in gene expression and abundance of CD4^+^ cells, with the transcriptome of Peripheral Blood Mononuclear Cells (PBMCs) reflecting CAD and diabetes mellitus (DM) [46]. These results suggest that peripheral blood has the potential to serve as a promising minimally invasive liquid biomarker for clinical diagnosis, aiding in the diagnosis and monitoring of atherosclerosis progression in different sexes and stages.

### Insights from liver and intestines

Through single-cell sequencing techniques, a new perspective on the complex cellular landscape of the aorta, especially in atherosclerotic areas, has confirmed that the accumulation of lipid intima is a key event in atherosclerosis caused by hypercholesterolemia [47]. Abnormal cholesterol accumulation in the body can lead to fatty liver lesions, and disrupted liver lipid metabolism further promotes risk factors for atherosclerosis, including inflammatory reactions, oxidative stress, insulin resistance, and foam cell formation [48–51]. As the primary organs involved in lipid metabolism, the liver and intestine play crucial roles in regulating circulating lipoproteins and cholesterol homeostasis [52–54]. Recent studies have also revealed a direct effect of intestinal flora on inflammation and lipid metabolism in the body [55, 56]. In this context, single-cell sequencing in the liver and intestines provides more direct evidence of atherosclerosis and contributes to a comprehensive understanding of its etiology and risk factors.

The liver and intestine exert influence on lipid metabolism and inflammation through various mechanisms, thereby impacting the occurrence and development of atherosclerosis [57–60]. Liver-derived single-cell RNA sequencing (scRNA-seq) data reveal specific enrichment of genes associated with serum lipid levels in hepatocytes [61]. Epigenetic and liposome analyses, combined with scRNA-seq, have shown that hepatocyte adenosine kinase (ADK) not only reduces liver Ppara expression and fatty acid oxidation but also promotes the pro-inflammatory activation of macrophages through ADK-driven liver cytokines [62, 63]. Additionally, a study by Oliver and colleagues analyzed different bone marrow cell clusters in non-alcoholic fatty liver disease (NAFLD) liver using scRNA-seq, demonstrating the adaptation of bone marrow cells in the liver to NAFLD progression and the unique inflammatory phenotype characterized by down-regulation of inflammatory calcium-binding protein (S100A8/A9) in macrophages and dendritic cell subsets [64]. These scRNA-seq findings highlight the role of the liver in lipid metabolism and inflammation and contribute to our understanding of potential mechanisms in atherosclerosis. On the other hand, recent studies have shown that the intestinal microbiota can regulate persistent gene expression, energy metabolism, and lipid metabolism, promoting the storage of absorbed energy in adipocytes [65–67]. Conversely, bile acids can induce changes in the intestinal microbiota and affect cholesterol synthesis. Zheng et al. [68] conducted an experiment in which mice were fed a high-fat diet or a standard diet, and the comparison of the two groups after two months revealed alterations in the intestinal flora through 16S rRNA gene sequencing. The high-fat diet group showed an increase in Bacteroides and thick-walled bacteria but a significant decrease in verrucous microflora, suggesting that bile acids can reduce cholesterol synthesis by reducing the abundance of related intestinal microbiota, thereby affecting the occurrence of atherosclerosis. Furthermore, the correlation analysis between metabonomics and macroeconomics indicates that the absence of PSRC1 can directly impact the intestinal microbiota and liver FMO3, thereby accelerating the production of TMAO and atherosclerosis [69]. In summary, single-cell analysis demonstrates the significant role of the liver and intestines in atherosclerosis.

With the wide application of single-cell sequencing and analysis techniques in the study of the structure and function of the liver, intestines, and intestinal microorganisms, new interventions to inhibit atherosclerosis will be discovered in the future. However, in eukaryotic samples such as hepatocytes, achieving full coverage of information-rich non-ribosomal transcripts can be accomplished by selectively initiating messenger RNA using oligonucleotides (dT) to exclude ribosomal RNA (rRNA) during cDNA synthesis. In contrast, bacterial mRNA lacks a poly(A) sequence, making the enrichment strategy unfeasible. Additionally, 16S and 23S rRNA sequences differ among species, posing challenges to general removal methods [70]. An available alternative strategy for bacterial rRNA removal is the depletion of abundant sequences by hybridization (DASH) technology. This approach utilizes programmed DNA cleavage by CRISPR-associated nuclease Cas9 to selectively remove "unwanted" fragments from the eukaryotic cDNA library with high sequence specificity [71]. DASH represents an attractive alternative to rRNA removal schemes due to its efficiency, high sensitivity, ease of implementation, and low cost, particularly for studies with limited material that have encountered difficulties with traditional ribose removal techniques [72].

## Opportunities for precision medicine

Atherosclerosis is a common chronic disease that imposes a significant financial burden on society. According to statistics, the national expenditure on atherosclerotic cardiovascular disease (ASCVD) in the United States reached $126 billion in 2015, and it is projected to increase to more than $309 billion by 2035 [73]. Furthermore, atherosclerosis is often accompanied by severe complications, which not only exacerbate the disease but also pose challenges for treatment [74]. Achieving precision medicine for atherosclerosis has long been the aim of clinicians and researchers.

Currently, research on atherosclerosis using single-cell profiling is still in the exploratory stage and has limited translation into clinical applications. Its primary focus is to re-examine and gain a better understanding of the disease process, as well as identify diagnostic and therapeutic markers. Harnessing the full potential of single-cell profiling will provide significant opportunities for atherosclerosis precision medicine. In the genomics era, the two clinical objectives are early diagnosis and early treatment. Early diagnosis and intervention in atherosclerosis can yield better outcomes by halting disease progression and improving the treatment response in patients with atherosclerosis.

### Early diagnosis of atherosclerosis

The early clinical manifestations of atherosclerosis are often concealed and easily overlooked, as the disease progresses for several years before causing obvious clinical symptoms [75]. Early and accurate diagnosis of atherosclerosis is expected to reduce the incidence of complications and mortality, which is the key to reduce potential clinical complications and cardio-cerebrovascular events. Early diagnosis of atherosclerosis involves a variety of methods. First of all, blood biochemical tests can help to known whether there are risk factors for the disease, so as to make a preliminary judgment; secondly, the scope and severity of the disease can be judged by ultrasonography, X-ray examination, electrocardiogram, coronary angiography and other examinations. Therefore, the economic burden of diagnosis of atherosclerosis is expensive [76].

Early and accurate diagnosis of atherosclerosis is crucial in reducing the incidence of complications and mortality, and it represents a key factor in minimizing potential clinical complications and cardio-cerebrovascular events. Intravascular ultrasound (IVUS) is a catheter-based intravascular imaging method, which can provide high-resolution cross-sectional images of coronary vessels in vivo. Widely used coronary artery lumen, vascular wall and atherosclerotic plaque formation [77, 78]is the most effective visualization method for early diagnosis of atherosclerosis [79–81]. However, IVUS not only requires skilled clinical skills and professional knowledge of image interpretation, but also brings certain risks to patients. In addition, its detection and quantification of specific plaque components have limitations, such as lipid-rich tissues and micronuclei [74, 82]. The above factors prevent IVUS from becoming the "gold standard" for the diagnosis of coronary atherosclerosis [83] and hinder its wide clinical use [84]. In contrast, single cell analysis, as one of the detection methods of early atherosclerosis, first of all, its examination trauma is less, which greatly reduces the risk of patients; secondly, single cell analysis can gain an in-depth understanding of cellular heterogeneity in complex tissues and can objectively and truly understand the progress of the disease. Finally, clinicians can simplify the generation and visualization of the results through software analysis [85] to reduce the difficulty of clinical application. These make single-cell analysis technology become one of the indispensable examination methods of early atherosclerosis.

However, with the advancement of single-cell analysis technology, researchers have begun to explore molecular-level solutions for early diagnosis of atherosclerosis. In 2018, Shayan et al. [86] analyzed monocyte expression in peripheral blood monocyte subsets from 253 young individuals, discovering that circulating endothelial cells (CECs) in peripheral blood can serve as a novel method to reflect molecular changes in the vascular endothelium. In 2022, Lin et al. [87] conducted a single-cell analysis of 45 human peripheral blood samples and identified specific peripheral immune cell subsets closely associated with the severity of coronary artery disease. By integrating immune and clinical features, they established a new cardiovascular disease (CVD) risk prediction model for stratifying patients at different stages of the disease. This non-invasive diagnostic method utilizing peripheral blood monocyte analysis represents a technological innovation for the early diagnosis of atherosclerosis [86].

Single-cell sequencing, used to screen differential genes in atherosclerosis cells, holds clinical significance for the early diagnosis of the disease. Clinical single-cell RNA sequencing analysis has confirmed CD68, PAM, and IGFBP6 genes as effective early diagnostic markers for unstable plaque [88]. It has also revealed the involvement of Tcf21 in the phenotypic regulation of smooth muscle cells in vivo, with the deletion of Tcf21 resulting in a decrease in fibrocytes within the protective fibrous cap [89]. Additionally, single-cell sequencing data has demonstrated a significant correlation between TSPAN4 expression and atherosclerotic regression of macrophages, intra-plaque bleeding, and plaque rupture [90]. These findings highlight CD68, PAM, IGFBP6, Tcf21, and TSPAN4 as potential target genes for the early detection of coronary artery disease. With the rapid development of various technologies such as single-cell analysis technology and GWAS [91–93], the cost of these technologies is on a downward trend, which makes the early diagnosis of atherosclerosis possible.

### Early treatment of atherosclerosis

In the process of early treatment for atherosclerosis patients, the exploration and improvement of therapeutic effects from different treatment modalities are essential. It is crucial for every clinical practitioner to fully understand the characteristics of various treatment methods. In this regard, single-cell analysis holds the potential to provide specific efficacy data on atherosclerosis, thereby facilitating the evaluation of early treatment options and assisting clinicians in making appropriate decisions.

Single-cell analysis enables the evaluation of drug efficacy by comparing changes in cell abundance and gene expression patterns before and after treatment in the same study subjects or different groups. For instance, in the case of desmosterol, an emerging immunomodulator for atherosclerosis lipid overload, single-cell analysis of anti-inflammatory macrophage markers' expression before and after treatment demonstrated its therapeutic efficacy in inhibiting inflammation by modulating macrophage cholesterol metabolism and inflammatory activation [94]. Similarly, RNA-seq analysis of macrophage phenotype and associated inflammatory genes in atherosclerotic samples from New Zealand rabbits revealed the pharmacokinetic advantage of genistein in the treatment of atherosclerosis [95]. Overall, in the context of atherosclerosis, single-cell sequencing tools offer clinicians the ability to sensitively assess early treatment at the single-cell level.

Plaque is a common clinical manifestation of atherosclerosis, leading to reduced blood supply and increasing the risk of heart attack, stroke, and even death [96, 97]. Plaque regression is an important clinical objective for reducing the burden of cardiovascular disease. The single-cell analysis technique reveals genes associated with plaque regression to ensure the effectiveness of clinical treatment. Single-cell analysis has demonstrated that tamoxifen-induced silencing of the Ntn1 gene alters the gene expression profile of plaque macrophages and reorganizes the immune cell landscape in the arterial wall, promoting atherosclerosis regression [98]. Furthermore, single-cell RNA sequencing data suggest that miR-33 is an important gene involved in the silent reprogramming of the immune cell landscape in atherosclerotic plaques, promoting atherosclerosis regression. Considering the growing interest in nanomedicine, numerous efficient nano-preparations have been utilized in the treatment of atherosclerosis. It is conceivable to combine genes that promote atherosclerosis plaque regression with nano-carriers through entrapment, encapsulation, adsorption, or covalent bonding to create a "nano-drug delivery system" for achieving effective early treatment of atherosclerosis [99].

### Fusion of multiple technologies for single cell sequencing

With the continuous advancement of sequencing technology, single-cell analysis is evolving towards the integration of multiple technologies. This provides a deeper understanding and characterization of the biological processes driving cell phenotypes. Combining single-cell analysis with other techniques and technologies, such as 3D cell culture and CRISPR/Cas9 gene editing, will further contribute to the accurate medical application of atherosclerosis.

The use of three-dimensional (3D) in vitro cell culture systems overcomes the limitations of two-dimensional (2D) cell cultures in replicating disease-related microenvironments and complex processes. This system facilitates the exploration of the etiology and pathogenesis of diseases. The combination of 3D cell culture and single-cell analysis enables more comprehensive screening of disease-related pathogenic genes and the development of individualized effective chemotherapeutic drugs for accurate patient treatment. For instance, RNASeg analysis of in vitro cultured pancreatic ductal gland (PDAC) cells identified potential biomarkers and revealed the synergistic antitumor effect of an epigenetic inhibitor and gemcitabine in pancreatic cancer cells [100]. The application of the three-dimensional in vitro cell culture system in atherosclerosis research is also progressing [101–104]. By integrating this system with single-cell sequencing, the goal of precision medicine can be achieved. Furthermore, CRISPR/Cas9, as an accurate, efficient, and convenient gene editing technology, realizes the precise modification of DNA sequences at the genome level by guiding RNA to recognize target gene sequence sites. It is fused with single-cell analysis to derive a variety of technologies, such as Perturb-Seq, CRISP-Seg, and Mosaic-Seg [105–108]. This fusion technology primarily uncovers potential therapeutic targets in atherosclerosis, such as the 15g26 gene in coronary artery disease and the ZEB2 gene [109, 110]. In the future, the integration of mature CRISPR/Cas9 gene editing technology with accessible sequencing techniques will facilitate whole-genome screening and expedite the development of precision medicine.

In theory, single-cell sequencing can provide answers to questions such as whether atherosclerosis has occurred, the disease stage of atherosclerosis, and the effectiveness of targeted drugs for atherosclerosis, all in one assay. However, practical and feasible clinical detection schemes are still being developed. In the future, the integration of appropriate technologies will make single-cell analysis not only affordable and user-friendly but also yield standardized results, enabling clinicians to interpret the data seamlessly.

### Road of clinical application

Although single-cell sequencing offers significant advantages in the study of atherosclerosis, there are also limitations and areas that require improvement. Firstly, single-cell sequencing technology still has a certain error rate, particularly in detecting low-expression genes, which can result in false negative or false positive results. This may affect the accuracy of detecting and analyzing important genes. Secondly, single-cell sequencing generates large amounts of data, necessitating substantial computing resources and specialized technical support for data storage, processing, and analysis. This restricts the widespread use of single-cell sequencing in clinical applications. Lastly, the cost of single-cell sequencing technology is high, and it requires complex operations and specialized training, which limits its adoption in clinical settings.

To address these challenges, several measures can be taken. Firstly, continuous efforts should be made to improve the accuracy and reliability of single-cell sequencing technology, develop more sensitive and precise detection methods, and reduce error rates. Secondly, collaboration with artificial intelligence can enhance research and application of single-cell sequencing by providing comprehensive data processing and analysis methods, thereby lowering the threshold for data processing and analysis [111]. For instance, artificial intelligence can leverage existing information in genome big data to ultimately deliver precise drugs [112]. Thirdly, increased investment in single-cell sequencing technology can help reduce its cost and promote its broader use in clinical applications. In conclusion, single-cell sequencing technology holds great potential and prospects for clinical applications in atherosclerosis. However, continuous efforts and improvements are needed to overcome its limitations and drawbacks. There is still progress to be made before single-cell sequencing becomes a routine tool in clinical practice.

## Conclusions

Single cell sequencing technology has successfully applied to study cell biological function in atherosclerosis with unprecedented high resolution. Future Multi-omics researches combining gene sequencing and phenotypic analysis will provide comprehensive images from the whole disease process of atherosclerosis. These data will enable people to clearly understand the molecular map of all stages of the disease and promote the development of accurate medical strategies in clinical treatment. First of all, the accurate analysis of the expression, localization information and functional changes of tens of thousands of genes in cells through single cell map technology is helpful to early screen the occurrence of atherosclerosis disease. Second, the real cell characteristics can be obtained by single cell analysis, which can provide independent information for rapid analysis and improve the quality and efficiency of disease treatment. Third, the study of single cell map can also provide strong support for the realization of individualized therapy because of the different cell state and expressed genes of each person.

In the process of accurate diagnosis and treatment of atherosclerosis, the target population of single cell analysis can include the following five categories of people. 1) Symptoms and consequences (sedentary, stress stress, sleep deprivation, etc.) considered as high-risk lifestyle factors [113]. 2) Patients who have been diagnosed with atherosclerosis, including but not limited to patients with atherosclerotic lesions in coronary arteries, carotid arteries, vertebral arteries, limb arteries and other parts. 3) Patients with suspected symptoms or signs of atherosclerosis, such as vascular stenosis, abnormal blood pressure, increased blood viscosity, etc. 4) Patients with risk factors for atherosclerosis [114], for example, high blood pressure, hyperlipidemia, diabetes, obesity, smoking and so on. 5) Patients who need to know the details of atherosclerotic lesions, such as patients who need to develop a more accurate treatment plan. It should be noted that although single cell sequencing is still in the research stage, it has not been widely used in clinical diagnosis. However, it does not prevent single cell sequencing from playing an important role in clinic now and in the future, and can assist clinical diagnosis, staging and necessary clinical differential diagnosis. Compared with the traditional blood test, ultrasound or imaging examination [115, 116], single cell sequencing has more advantages in the examination, which can better distinguish the images overlapped by the previous routine examination and better distinguish the lesions. To clarify the relationship between plaques and surrounding tissues, but also can well evaluate the progress of plaques, so it plays an irreplaceable role.

Generally speaking, the emergence of single-cell analysis technology has greatly expanded our understanding of atherosclerosis and promoted the development of precise medical strategies in clinical treatment. With the continuous progress of single-cell analysis technology, this will contribute to the accurate medical management of atherosclerosis. However, there are still many problems to be considered about the application of single cell analysis in atherosclerosis species. For example, the new experimental data obtained from the study of atherosclerosis by single cell analysis technology still need to be further verified, including new cell types and new therapeutic targets, and how to establish the correlation between single cell sequencing data and traditional clinical examination techniques in the early diagnosis of atherosclerosis. Besides, the research of the combination of single cell analysis technology and other existing technologies still needs to be supported by more exquisite experiments. We still have a long way to go in the future, hoping to better lay the foundation for accurate diagnosis and treatment management of atherosclerosis. In conclusion, this review summarizes the research status of single cell sequencing technology in atherosclerotic plaque, blood, liver and intestine, and describes its clinical application to atherosclerosis. Combining single cell sequencing technology with other technologies can promote more accurate medical intervention. In the future, the correlation between single-cell sequencing data and a large number of atherosclerosis clinical parameters will be gradually established to achieve clinical application transformation. We expect its widespread use to bring good news to the precision medicine of atherosclerosis.

## Data Availability

Not applicable.

## Abbreviations

CAD: Coronary artery disease
CVD: Cardiovascular disease
ASCVD: Atherosclerotic cardiovascular disease
DM: Diabetes mellitus
ADK: Adenosine kinase
NAFLD: Non-alcoholic fatty liver disease
DNMT3A: Recombinant DNA Methyltransferase 3A
TET2: Tet oncogene family member 2
ASXL1: Additional sex comb-like 1
JAK2: Janus kinase 2
MACS: Magnetically activated cell sorting
FACS: Fluorescence-activated cell sorting
TREM2: Bone marrow cell 2
VSMCs: Vascular smooth muscle cells
PBMCs: Peripheral Blood Mononuclear Cells
CECs: Circulating endothelial cells
3D cells: Three-dimensional cells
MACS: Magnetically activated cell sorting
FACS: Fluorescence-activated cell sorting
GWAS: Genome-wide association studies
scRNA-seq: Single-cell RNA sequencing
